# Levels of essential and non-essential metals in ginger (*Zingiber officinale*) cultivated in Ethiopia

**DOI:** 10.1186/s40064-015-0899-5

**Published:** 2015-03-04

**Authors:** Yohannes Wagesho, Bhagwan Singh Chandravanshi

**Affiliations:** Department of Chemistry, College of Natural Sciences, Addis Ababa University, P.O. Box 1176, Addis Ababa, Ethiopia

**Keywords:** Ginger, *Zingiber officinale*, Essential metals, Non-essential metals, Soil, Ethiopia

## Abstract

**Background:**

Ginger (*Zingiber officinale* Roscoe) is a common condiment for various foods and beverages and widely used worldwide as a spice. Its extracts are used extensively in the food, beverage, and confectionary industries in the production of products such as marmalade, pickles, chutney, ginger beer, ginger wine, liquors, biscuits, and other bakery products. In Ethiopia, it is among the important spices used in every kitchen to flavor stew, tea, bread and local alcoholic drinks. It is also chiefly used medicinally for indigestion, stomachache, malaria, fevers, common cold, and motion sickness. The literature survey revealed that there is no study conducted on the determination of metals in ginger cultivated in Ethiopia. Hence it is worthwhile to determine the levels of essential and non-essential metals in ginger cultivated in Ethiopia.

**Methods:**

The levels of essential (Ca, Mg, Fe, Zn, Cu, Co, Cr, Mn, and Ni) and non-essential (Cd and Pb) metals in ginger (*Zingiber officinale* Roscoe) cultivated in four different regions of Ethiopia and the soil where it was grown were determined by flame atomic absorption spectrometry. 0.5 g of oven dried ginger and soil samples were digested using 3 mL of HNO_3_ and 1 mL of HClO_4_ at 210°C for 3 h and a mixture of 6 mL aqua-regia and 1.5 mL H_2_O_2_ at 270°C for 3 h, respectively.

**Results:**

The mean metal concentration (μg/g dry weight basis) ranged in the ginger and soil samples, respectively, were: Ca (2000–2540, 1770–3580), Mg (2700–4090, 1460–2440), Fe (41.8–89.0, 21700–46900), Zn (38.5–55.2, 255–412), Cu (1.1–4.8, 3.80–33.9), Co (2.0–7.6, 48.5–159), Cr (6.0–10.8, 110–163), Mn (184–401, 1760–6470), Ni (5.6–8.4, 14.1–79.3) and Cd (0.38–0.97, 0.24–1.1). The toxic metal Pb was not detected in both the ginger and soil samples.

**Conclusion:**

There was good correlation between some metals in ginger and soil samples while poor correlation between other metals (Fe, Ni, Cu). This study revealed that Ethiopian gingers are good source of essential metals and free from toxic metal Pb while containing negligible amount of Cd.

## Introduction

The delightful flavour and pungency of spices make them indispensable in the food industry to flavour, improve and increase the appeal of their products. Spices impart aroma, colour and taste to food preparations and sometimes mask undesirable odours. In addition, they are reputed to possess several medicinal and pharmacological properties and hence find position in the preparation of a number of medicines (Parthasarathy et al. 2008).

Ethiopia is among the largest consumer of spices in Africa. The major use of spices in the country is in the preparation of a highly spiced stew known as 'Wot' which together with 'Injera' is consumed by a large proportion of the population everyday as their main food. In addition, spices are also used by the numerous ethnic groups in the country to flavor bread, meat, soups, different vegetables, and as medicines and perfumes (Asfaw and Demissew 2009).

The spice ginger obtained from the underground stems or the rhizome of *Zingiber officinale* Roscoe, one of the most widely used species of the family Zingiberaceae, is a common condiment for various foods and beverages. Both fresh and dried ginger rhizomes are used worldwide as a spice, and ginger extracts are used extensively in the food, beverage, and confectionary industries in the production of products such as marmalade, pickles, chutney, ginger beer, ginger wine, liquors, biscuits, and other bakery products (Mishra 2009). Ginger is also widely used in both traditional and contemporary natural medicine (Zingiber officinale 2010). In Ethiopia, it is among the important spices used in every kitchen to flavor stew, tea, bread and local alcoholic drinks (Asfaw and Abegaz, 1995). It is also chiefly used medicinally for indigestion, stomachache, malaria, fevers, common cold, and motion sickness.

The major ginger growing area in Ethiopia includes wetter regions at altitude below 2000 m in Kefa, Illubabur, Gamo Gofa, Sidama, Wellega, Wolaita, and Kembata-Tambaro. Currently, it has become an important cash crop for farmers in southern and south-western parts of Ethiopia. The production of this spice has been expanding in most parts of the country, as it can be grown under varied climatic conditions. It thrives well in areas with altitudes from sea level to 1500 m, mean annual temperature of 20–32°C and with total rainfall greater than 1200 mm. Well-drained, fertile and friable soil with enough humus and neutral pH is the ideal soil type for the production of ginger (Asfaw and Demissew 2009; Hailemicheal et al. 2008).

The unique flavor properties of ginger arise from the combination of pungency and aromatic essential oil. The main pungent compounds in fresh ginger are a series of homologous phenolic ketones known as gingerols. The gingerols are thermally unstable and are converted under high temperature to shogaol (Mishra 2009). Shogaols, which are more pungent than gingerols, are the major pungent compounds in dried ginger (Jolad et al. 2004).

The ginger rhizome also contains resin, proteins, cellulose, pentosans, starch and mineral elements. Of these, starch is the most abundant and comprises 40–60% of the rhizome on a dry weight basis. The relative abundance of certain constituents of ginger is determined by the cultivar grown, the environmental conditions of growth and the stage of maturity at harvest (Parthasarathy et al. 2008).

Humans require a suite of mineral elements in varying amounts for proper growth, health maintenance and general well being. Minerals are essential components of our diet that serve as cofactors in the thousands of enzyme-controlled reactions, control the action of nerves and muscles, help maintain the body's water balance, and buffer the pH (acidity) of the cell and extracellular fluids (Minerals-Learn 2010). Plant-derived foods have the potential to serve as dietary sources for all human-essential minerals that make a significant contribution to daily mineral needs at all stages of the life cycle (Grusak 2002). Generally, too low or too high of a concentration of trace elements in human diet can affect the quality of human life.

Mineral uptake by plants can be affected by several factors including mineral concentrations in soils, soil pH, cation exchange capacity, organic matter content, types and varieties of plants, and age of the plant (Jung 2008). In ideal word we would take in our daily requirement of minerals by eating plants that grow in mineral rich soils (Harold and Leslie 2000; Lokeshwari and Chandrappa 2006). The contamination of soil by atmospheric deposition of toxic metals affects soil properties and further increases plant metal levels through root uptake (Pandey and Pandey 2009), and eventually these metals are taken up by plants parts and transfer some into the food chain. Consequently, higher soil heavy metal concentration can result in higher levels of uptake by plants (Ebong et al. 2008).

Contamination and subsequent pollution of the environment by toxic heavy metals have become an issue of global concern due to their sources, widespread distribution and multiple effects on the ecosystem. Heavy metals are generally present in agricultural soils at low levels. Due to their cumulative behaviour and toxicity, however, they have a potential hazardous effect not only on crop plants but also on human health (Uwah et al. 2009). Therefore, a comprehensive study related to the assessment of levels of essential and toxic metals of plants and soil where the plant has grown is crucial with respect to human health and the quality of its products.

Some studies have been carried out on levels of essential and non-essential metals in some spices and medicinal plants cultivated in Ethiopia (Derbie and Chandravanshi 2011; Gebre and Chandravanshi 2012; Kitata and Chandravanshi 2012; Weldegebriel et al. 2012; Aregahegn et al. 2013; Mekebo and Chandravanshi 2014; Endalamaw and Chandravanshi 2015; Dubale et al. 2015). Some studies have also been conducted on determination of essential and non-essential metal levels of ginger in Nigeria (Obiajunwa et al. 2002; Ogunwandea and Olawore 2004; Aiwonegbe and Ikhuoria 2007), India (Devi et al. 2008), and Saudi Arabia (Al-Eed et al. 2002; Alwakeel 2008). However, the literature survey revealed that there is no study conducted on the determination of metals in ginger cultivated in Ethiopia. Since ginger is used as a spice for many peoples of Ethiopia and it is cash crop, the knowledge of their mineral levels is of particular interest. Hence it is worthwhile to determine the levels of essential and non-essential metals in ginger cultivated in Ethiopia. Therefore, this study deals with assessment of levels of metals (essential and non-essential) in ginger cultivated in Ethiopia and it aims to fill the gap at least partially in the area and initiate others for further study on ginger and closely related plants widely used throughout the country. The outcome of this study will ultimately help to ensure the dietary safety of the society and improving the country's economy by increasing both quality and quantity of ginger cultivated in Ethiopia.

The aim of this study was to determine the levels of selected major, trace and toxic metals (Ca, Mg, Fe, Zn, Cu, Co, Cr, Mn, Ni, Cd, and Pb) in ginger cultivated in Ethiopia, to assess the level of minerals in soil samples where the ginger was grown and to correlate the levels of minerals in the ginger with that of soil in which it was cultivated. It was also aimed to compare the levels of metals in the ginger cultivated in Ethiopia with the levels of metals in the ginger from other countries.

## Methods

### Equipments and reagents

Chopping board (PTFE, China) and Teflon (PTFE) knife were used to cut ginger rhizome in to pieces. A drying oven (Digitheat, J.P. Selecta, Spain) was used to dry ginger sample. Electronic blending device (Moulinex, France) was used for grinding and homogenizing the sample to determine the total metal content of the ginger. Mortar and pestle was used to grind soil sample. Analytical balance (Larko, LA114, 110 g/0.1 g) with precision of ± 0.0001 g was used to weigh the ginger and soil sample. A 100 mL round bottomed flasks fitted with reflux condensers were used in Kjeldahl apparatus hot plate to digest the dried and powdered ginger and soil samples. Borosilicate volumetric flasks (25, 50 and 100 mL) were used during dilution of sample and preparation of metal standard solutions. Measuring cylinders (Duran, Germany), pipettes (Pyrex, USA), and micropipettes (Dragonmed, 1–10 μL, 100–1000 μL, Shangai, China) were used during measuring different quantities of volumes of sample solution, acid reagents and metal standard solutions. Flame atomic absorption spectrophotometer, FAAS (Buck Scientific Model 210VGP AAS, East Norwalk, USA) equipped with deuterium arc back ground correctors and hollow cathode lamps with air-acetylene flame was used for the determination of the metals (Ca, Mg, Cu, Zn, Mn, Ni, Fe, Co, Cr, Pb and Cd) in the ginger and soil samples.

All the reagents used were of analytical grade. HNO_3_ (69–72%) and HClO_4_ (70%) (Research Lab Fine Chem Industries, Mumbai, India) were used for the digestion of ginger samples. Aqua-regia prepared from 3:1 ratio of 37% HCl (Riedel-deHaën, Germany) and (69–72%) HNO_3_, and extra pure hydrogen peroxide 30% H_2_O_2_, (Scharlau, European Union), were used for the digestion of soil sample. Lanthanum nitrate hydrate (98%, Aldrich, USA) was used to avoid refractory interference (for releasing calcium and magnesium from their phosphates). Stock standard solutions containing 1000 mg/L, in 2% HNO_3_, of the metals Ca, Mg, Cu, Zn, Mn, Ni, Fe, Co, Cr, Pb and Cd (Buck Scientific Puro-Graphic, USA) were used for preparation of calibration standards and in the spiking experiments. Deionized water (chemically pure with conductivity 1.5 μs/cm and below) was used for dilution of sample and intermediate metal standard solutions prior to analysis and rinsing glassware and sample bottles.

### Sampling

Samples were collected from four major ginger producing areas in Ethiopia namely Tepi, Bombae, Hadaro and Illubabur. Tepi is located in the Sheka zone, Southern Nations, Nationalities and Peoples' Region (SNNPR), 596 km southwest of Addis Ababa with an elevation of 1097 m above sea level and lies between 7°12’–7°89’N and 35°24’ to 37°90’E. The annual mean temperature of the area ranges between 15.1–27.5°C and the annual mean rainfall ranges 1201–1800 mm. Bombae is located in Wolayita Zone, SNNPRS, 390 km south west of Addis Ababa, lies between 6°51’ and 7°35’N and 37°46’–38°1’E. Hadaro is located in Kembata zone of SPNNR. It is 270 km far from the capital city of Ethiopia which is Addis Ababa. Illubabor is located in the Oromia Region. It has a latitude and longitude of 8°18′N and 35°35′E ﻿and an altitude of 1605 m.

Fresh ginger samples were collected from the farmlands of four areas in southern and south western Ethiopia particularly Illubabur (Oromia region), Tepi (Sheka, SNNPR), Hadaro (Kambata, SNNPR), and Bombae (Wolayita, SNNPR). These sampling sites were selected based on large-scale production area of ginger in the country so that the sample partly represents the whole ginger cultivated in Ethiopia. To collect the representative sample from each sampling sites, three sub-samples (500 g each) were taken from farmlands which were roughly two km away from each other. These farmlands were randomly chosen from the three triangular corners of the area. Half kilogram of fresh ginger samples were collected from each farmland and put in clean polyethylene plastic bags labelled and brought to the laboratory for further pre-treatment. The three sub-samples were mixed together after grinding in a blender to homogenize and form bulk samples that represent each sampling areas. Finally four ginger bulk samples one from each stated areas were prepared for analysis. 12 samples with 0.5 g aliquot (three from each bulk sample) were taken for final digestion.

For comparative analysis of mineral levels in ginger and the soil where the ginger was cultivated, the soil samples were collected from the surface horizon (15–20 cm) depth of the same four sampling areas of ginger. Sampling was done similar to ginger. Half-kilogram soil samples were collected from each farmland. The three sub-samples were mixed together after grinding using a mortar and pestle to homogenize and form bulk samples that represent each sampling areas. Finally four soil bulk samples one from each stated areas were prepared for analysis. 12 samples with 0.5 g aliquot (three from each bulk sample) were taken for final digestion.

### Sample preparation

Fresh ginger collected from the sampling areas was kept in plastic bags and transported to the laboratory. The rhizomes were washed with a running tap water so as to remove adsorbed soil particulates and then rinsed with deionized water. The thin outer skin of ginger was removed by plastic knife and chopped in to pieces nearly uniform size to facilitate drying uniformly. The sample was exposed to sun light for two days to reduce the moisture content. Then, to have constant mass, the sample was oven dried at 80°C for 24 h so as to express the result in terms of dry mass basis. The dried ginger was powdered using electronic blender and sieved to prepare fine powder of ginger for digestion.

The soil sample collected from the four sampling area were air dried to constant weight for three days and sieved through a 2-mm polyethylene sieve to remove large debris, stones, and pebbles. Then, the samples were ground using a mortar and pestle to pass a 500-μm sieve, homogenized, and ready for digestion.

### Moisture content of ginger

To determine the moisture content of fresh ginger, first it was carefully washed with tap water to remove adsorbed soil particulates and exposed to air to vaporize water on the surface of it. Then it was weighed with electronic balance to record the initial weight with its moisture content. After oven drying at 80°C for two days it was re-weighed and re-dried until it gave constant mass. The moisture content of four samples was between 75.0–84.9%. Therefore, fresh ginger cultivated in Ethiopia has comparable moisture content with the value reported by Govindarajan (1982), which is 80.9% for typical analysis of market sample of ginger.

### Digestion of ginger and soil samples

The basic requirements for sample preparation for analysis are to get an optimum condition for digestion. The optimum condition is the one which required minimum reagent volume consumption, minimum reflux time, clarity of digests, and ease of simplicity (Huang et al. 2004; Wilson et al. 2005; Demirel et al. 2008; Chen and Ma 2001; Gaudino et al. 2007; Endalamaw and Chandravanshi 2015).

In this study, to prepare a clear colorless sample solution that is suitable for the analysis using FAAS, different ginger digestion procedures were optimized using the HNO_3_ and HClO_4_ acid mixtures by varying parameters such as volume of the acid mixture, digestion time and digestion temperature (Huang et al. 2004; Wilson et al. 2005; Demirel et al. 2008). The results are given in Table 1. From the optimization procedures, the acid mixture of 3 mL of HNO_3_ (69–70%) and 1 mL of HClO_4_ (70%), digestion time of 3 h and digestion temperature of 210°C were found the optimal condition for 0.5 g ginger sample. These optimum conditions were selected based on clarity of digests, minimum reagent volume consumption, minimum digestion time, simplicity and minimum temperature applied for complete digestion of sample.

**Table 1:** **Different conditions tested for optimization of digestion procedure for 0.5 g ginger samples**

**Trial no.**	**Reagent(s) used**	**Reagent volume (mL)**	**Temp. (°C)**	**Digestion time (h)**	**Results**
Optimization for reagent volume					
1	HNO_3_:HClO_4_	3:3	270	3:00	Deep yellow
2	HNO_3_:HClO_4_	4:2	270	3:00	Yellow
3	HNO_3_:HClO_4_	3:2	270	3:00	Clear yellow
4	HNO_3_:HClO_4_	4:1	270	3:00	Almost clear
5	HNO_3_:HClO_4_	2:2	270	3:00	Clear light yellow
**6**	**HNO**_**3**_ **:HClO**_**4**_	**3:1**	**270**	**3:00**	**Clear and colourless**
Optimization for temperature					
1	HNO_3_:HClO_4_	3:1	150	3:00	Deep yellow
2	HNO_3_:HClO_4_	3:1	180	3:00	Light yellow
**3**	**HNO**_**3**_ **:HClO**_**4**_	**3:1**	**210**	**3:00**	**Clear and colourless**
4	HNO_3_:HClO_4_	3:1	240	3:00	Clear and colourless
5	HNO_3_:HClO_4_	3:1	270	3:00	Clear and colourless
6	HNO_3_:HClO_4_	3:1	300	3:00	Clear and colourless
Optimization for digestion time					
1	HNO_3_:HClO_4_	3:1	210	1:45	Deep yellow
2	HNO_3_:HClO_4_	3:1	210	2:00	Light yellow
3	HNO_3_:HClO_4_	3:1	210	2:15	Light yellow
4	HNO_3_:HClO_4_	3:1	210	2:30	Clear light yellow
5	HNO_3_:HClO_4_	3:1	210	2:45	Clear and colourless
**6**	**HNO**_**3**_ **:HClO**_**4**_	**3:1**	**210**	**3:00**	**Clear and colourless**

Rows with bold font indicate the optimal condition for the given parameter.

A modified aqua-regia (HNO_3_ + HCl + H_2_O_2_) was selected as digestion reagent for soil sample digestion in this work (Chen and Ma 2001; Wilson et al. 2005; Gaudino et al. 2007). The optimum conditions (Table 2) for soil sample digestion were a reagent mixture of 6 mL aqua-regia (3:1 ratio of HCl to HNO_3_) and 1.5 mL H_2_O_2_, digestion temperature of 270°C and digestion time of 3 h for 0.5 g soil sample.

**Table 2:** **Different conditions tested for optimization of digestion procedure for 0.5 g soil samples**

**Trial no.**	**Reagent(s) used**	**Reagent volume (mL)**	**Temp. (°C)**	**Digestion time (h)**	**Results**
Optimization for reagent volume					
1	Aqua-regia:H_2_O_2_	6.5:1	300	3:00	Deep yellow with suspension
**2**	**Aqua-regia:H**_**2**_ **O**_**2**_	**6:1.5**	**300**	**3:00**	**Light yellow with no suspension**
3	Aqua-regia:H_2_O_2_	5.5:2	300	3:00	Light yellow with no suspension
Optimization for temperature					
1	Aqua-regia:H_2_O_2_	6:1.5	240	3:00	Deep yellow with suspension
**2**	**Aqua-regia:H**_**2**_ **O**_**2**_	**6:1.5**	**270**	**3:00**	**Light yellow with no suspension**
3	Aqua-regia:H_2_O_2_	6:1.5	300	3:00	Light yellow with no suspension
Optimization for digestion time					
1	Aqua-regia:H_2_O_2_	6:1.5	270	2:00	Deep Yellow with suspension
2	Aqua-regia:H_2_O_2_	6:1.5	270	2:30	Light yellow with suspension
**3**	**Aqua-regia:H**_**2**_ **O**_**2**_	**6:1.5**	**270**	**3:00**	**Light yellow with no suspension**

Rows with bold font indicate the optimal condition for the given parameter.

Applying the optimized condition, 0.5 g of dried and homogenized ginger samples were transferred into a 100 mL round bottomed flask. Then 4 mL of a mixture of HNO_3_ (69–72%) and HClO_4_ (70%) with a volume ratio of 3:1 (v/v) was added and the mixture was digested on a Kjeldahl digestion apparatus fitting the flask to a reflux condenser by setting the temperature first at 120°C for 30 min and then increased to 210°C for the remaining 2 h and 30 min. The digest was allowed to cool to room temperature for 10 min without dismantling the condenser from the flask and for 10 min after removing the condenser. To the cooled solution 15 mL of deionized water was added to dissolve the precipitate formed on cooling and to minimize dissolution of filter paper by the digest residue while filtering with Whatman, (110 mm, diameter), filter paper into 50 mL volumetric flask. The round bottom flask was rinsed subsequently with 5 mL deionized water until the total volume reached around 45 mL. To this final solution, 3 mL lanthanum nitrate solution (1% w/w) was added and the solution was filled to the mark (50 mL) with deionized water. The digestion was carried out in triplicate for each bulk sample. Digestion of a reagent blank was also performed in parallel with the ginger samples keeping all digestion parameters the same. The digested samples were kept in the refrigerator, until the levels of all the metals in the sample solutions were determined by FAAS.

Applying the optimized condition, 0.5 g of dried and homogenized soil samples were transferred into a 100 mL round bottomed flask. To this 6 mL of aqua-regia (3:1 ratio of 37% HCl to (69–72%) HNO_3_, respectively) and followed by 1.5 mL of 30% H_2_O_2_ were added and the mixture was digested on a Kjeldahl digestion apparatus fitting the flask to a reflux condenser by setting the temperature first at 180°C for the first 30 min and then raised to 240°C for the next 30 min and finally raised to 270°C for the remaining 2 h. The rest steps were similar for both ginger and soil sample digestion procedure.

### Instrument calibration

Calibration metal standard solutions were prepared for each of the metals from an intermediate standard solution containing 10 mg/L which was prepared from the atomic absorption spectroscopy standard stock solutions that contained 1000 mg/L. These intermediate standards were diluted with deionized water to obtain four working standards for each metal of interest. Then, Ca, Mg, Cu, Zn, Mn, Ni, Fe, Co, Cr, Pb and Cd were analyzed with FAAS (Buck Scientific Model 210GP) equipped with deuterium arc background corrector and standard air-acetylene flame system using external calibration curve. Three replicate determinations were carried out on each sample. All the eleven metals were determined by absorption/concentration mode and the instrument readout was recorded for each solution manually. The same analytical procedure was employed for the determination of elements in digested blank solutions.

The instrument was calibrated using four series of working standards. The working standard solutions of each metal were prepared freshly by diluting the intermediated standard solutions (10 mg/L). The correlation coefficients of the calibration curves were > 0.9999 which confirmed an excellent correlation between the absorbance and the concentration. The method detection limits were in the range 1–5 μg/g dry weight for the ginger and soil samples which indicated that the FAAS method used are applicable to detect the presence of metals in the ginger and soil samples at trace levels.

### Method performance and method validation

The criteria used for evaluating analytical methods are called figures of merit. Based on these characteristics, one can predict whether a method meets the needs of intended purpose. These figures of merit are accuracy, precision, sensitivity, detection limits, and the quantitation limits (Mitra 2003).

In this study, the precision of the results were evaluated by the pooled standard deviation, and relative standard deviation of the results of nine measurements for a given bulk sample (i.e. three samples (n = 3) and triplicate readings for each sample).

In the present study, method detection limit for each metal was estimated by digesting six analytical blanks with the optimized procedure for both ginger and soil samples. Triplicate analyses of six blank samples for all elements were performed and the pooled standard deviation of the six blank reagents was calculated. The detection limits were obtained by multiplying the pooled standard deviation of the reagent blank (S_blank_) by three (MDL = 3 x S_blank_, n = 6). The method detection limit of each metal was ≤ 5 μg/g which indicated that the method was applicable to the determination of metals at trace levels in both the ginger and soils samples.

In this work, the method validation was established by spiking experiments. The spiked samples were prepared by adding a small known quantity of metal standard solutions. For spiking ginger sample, 200 μL of 1000 mg/L Ca, 300 μL of 1000 mg/L Mg, 12.5 μL of 1000 mg/L Zn, 75 μL of 1000 mg/L Mn, 31.5 μL of 40 mg/L Ni, 10 μL of 1000 mg/L Fe, 43.5 μL of 40 mg/L Co, 25 μL of 40 mg/L Cu, 62.5 μL of 40 mg/L Cr and 30 μL of 10 mg/L Cd standard solutions were added to round bottomed flask (100 mL) containing 0.5 g ginger sample. For soil sample spiking, 350 μL of 1000 mg/L Ca, 250 μL of 1000 mg/L Mg, 235 μL of 1000 mg/L Fe, 65 μL of 1000 mg/L Mn, 75 μL of 1000 mg/L Zn, 20 μL of 1000 mg/L Ni, 200 μL of 40 mg/L Cu, 40 μL of 1000 mg/L Co, 35 μL of 1000 mg/L Cr and 32 μL of 10 mg/L Cd standard solutions were added to round bottomed flask (100 mL) containing 0.5 g soil sample. The spiked and non-spiked samples were digested and analysed in similar condition.

The results of percentage recoveries for the studied metal nutrients in both ginger and soil samples were within the acceptable range (93–106%) in the ginger and (93–107%) in the soil samples. These results verified that the optimized digestion procedure was valid for both ginger and soil sample analysis.

### Statistical analysis

The analysis of variance for the equality of means and correlation between the elements in ginger and soil samples were done using the SPSS 16.0.

## Results

The concentration of eleven elements (Ca, Mg, Fe, Zn, Cu, Co, Cr, Mn, Ni, Cd, and Pb) in the digested samples of ginger and soil were determined by FAAS. Among the determined metals Pb was below the method detection limit (0.002 mg/g in the ginger and 0.004 mg/g in the soil) and the concentration of the rest of metals are shown with their respective standard deviation in Tables 3 and 4. The most abundant metal among the macro-elements in ginger was Mg followed by Ca whereas Mn content was the predominant among the tested micronutrient heavy metals followed by Fe, Zn, Co and Cu. In soil sample the most abundant metal was Fe followed by Mn, Ca, Mg, Zn, Cr, Co, Cu, Ni, and Cd.

**Table 3:** **Average concentration (mean ± SD, n = 3, μg/g dry weight basis) of major, trace and toxic metals in ginger samples from Tepi, Bombae, Hadaro and Illubabur**

**Metal**	**Average concentration (mean ± SD, n = 3, μg/g dry weight basis)**			
	**Tepi ginger**	**Bombae ginger**	**Hadaro ginger**	**Illubabur ginger**
**Ca**	2000 ± 47	2540 ± 93	2190 ± 24	2490 ± 41
**Mg**	2990 ± 9	2700 ± 57	2760 ± 11	4090 ± 105
**Cu**	4.78 ± 0.34	1.86 ± 0.18	2.53 ± 0.19	1.10 ± 0.05
**Zn**	55.2 ± 3.9	39.6 ± 0.5	38.5 ± 0.5	54.0 ± 2.7
**Mn**	385 ± 9	285 ± 4.3	184 ± 3.6	401 ± 12
**Ni**	5.61 ± 0.44	5.46 ± 0.48	6.78 ± 0.53	8.40 ± 0.32
**Fe**	44.2 ± 3.3	55.4 ± 5.0	41.8 ± 2.8	89.0 ± 6.1
**Co**	7.58 ± 0.46	5.68 ± 0.40	2.04 ± 0.14	2.18 ± 0.18
**Cr**	9.28 ± 0.61	6.02 ± 0.14	9.17 ± 0.62	10.8 ± 0.2
**Cd**	0.97 ± 0.08	0.38 ± 0.02	0.38 ± 0.02	0.70 ± 0.07
**Pb**	ND	ND	ND	ND

ND: Concentration of the tested heavy metal was below the method detection limit.

**Table 4:** **Average concentration (mean ± SD, n = 3, μg/g dry weight basis) of major, trace and toxic elements in soil samples from Tepi, Bombe, Hadaro and Illubabur**

**Metal**	**Average concentration (mean ± SD, n = 3, μg/g dry weight basis)**			
	**Tepi soil**	**Bombae soil**	**Hadaro soil**	**Illubabur**
**Ca**	3580 ± 16	2060 ± 10	2040 ± 43	1770 ± 39
**Mg**	2430 ± 141	1460 ± 45	1660 ± 8	2440 ± 8
**Cu**	33.9 ± 0.5	3.76 ± 0.07	6.77 ± 0.17	33.7 ± 0.8
**Zn**	389 ± 36	344 ± 28	255 ± 14	413 ± 39
**Mn**	6470 ± 81	1760 ± 26	1920 ± 28	4680 ± 32
**Ni**	79.3 ± 1.2	14.1 ± 0.3	21.4 ± 1.0	73.1 ± 4.7
**Fe**	46900 ± 600	21800 ± 821	21700 ± 407	46170 ± 484
**Co**	159 ± 2.8	57.1 ± 2.1	48.5 ± 0.6	132 ± 1.9
**Cr**	139 ± 12	110 ± 7.6	114 ± 1.8	163 ± 2.5
**Cd**	1.08 ± 0.08	0.24 ± 0.02	0.73 ± 0.06	1.20 ± 0.07
**Pb**	ND	ND	ND	ND

ND: Concentration of the tested heavy metal was below the method detection limit.

## Discussion

### Distribution pattern of the metals in ginger samples

Mineral uptake in plants is a function of mineral concentrations in soils, soil pH, cation exchange capacity, organic matter content, types and varieties of plants, and age of the plant (Jung 2008). As it can be seen from Table 3, there is large difference in concentration of different metals within ginger sample and slight variation in metals of the same type along with geographical location. The range of concentration (in mg/kg) pattern of elements in ginger sample collected from four sampling area is given in Table 3.

Ginger contained higher amount of Mg (2700–4090 mg/kg), followed by Ca (2000–2540 mg/kg). The higher levels of Mg in the ginger is probably due to the fact that nutrient elements such as N, P, K, S, and Mg are highly mobile in the plant tissue and trans-located from old plant tissue to new plant tissue. The other probable reason for higher concentration of Mg and Ca is if the soil which have been used for cultivating the plant, are highly fertilized with manure and organic residues, K, Ca and Mg are highly available for plant uptake. Hence, the plant has high amount of these metals.

Mn (184–401 mg/kg) was the most accumulated trace metal followed by Fe (41.8–89.0 mg/kg) and Zn (38.5–55.2 mg/kg) in ginger sample. Higher Mn levels in the ginger may be attributed to the availability of this micronutrient heavy metal in relatively acidic soils of the farmland. The chemical forms of Mn present in soil are known to depend on soil pH. In acidic soil, the easily absorbed form, Mn^+2^ released from soil by H^+^, which is produced from NH_4_^+^ (Zubillaga et al. 2008), can be readily taken up and accumulated in the ginger.

It has been reported that Fe and Zn are the main elements that plant could accumulate and pass up in the food chain. Therefore, the high concentration of Zn from trace metals next to Mn and Fe in ginger may be because of the fact that these ions are readily transferred from the soil to plants, and accumulated in plants.

The levels of other essential trace metals detected in ginger were Co (2.04–7.58 mg/kg), Ni (5.61–8.40 mg/kg), Cr (6.02–10.8 mg/kg) and non-essential heavy metals Cd (0.38–0.97 mg/kg). The level of Cd was the least among the metals; however due to its toxicity deserves special concern. The non-essential heavy metal, Pb, was found to be below the method detection limit. In general the concentration pattern of metals in ginger was decreased as Mg > Ca > Mn > Fe > Zn > Cr > Ni > Co > Cu > Cd.

As it can be seen from Table 3, ginger are a good source of major and trace metals that are essential to human in addition to its food flavouring purpose. The small amount of Co and Cu found in ginger does not contradict with the requirement of the metal for proper functioning of the body, because these metals are required in small amount (Co = 0.3 mg/day as a constituent of vitamin B_12_ and Cu = 3.5 mg/day).

### Daily intake of metals from ginger

Daily intake of metals from ginger has been calculated based on the assumption that an adult person consumes 5 g fresh ginger per day in different form. The moisture content of Ethiopian fresh ginger was found in the range 75–85% with a mean value of 80%. Thus the dry mass of 5 g ginger is 1 g. The metal contents of 1 g dry ginger are given in Table 5. For comparison the recommended or adequate daily intake and the allowable upper limit of daily intake of metals are also given in the Table 5. The data in the table clearly show that the daily intake of Ca, Mg and Cu are well below the recommended daily intake while Zn, Mn, Fe, and Cr exceed the recommended daily intake but are well below the allowable upper limit of daily intake of these metals. Daily intake of Ni and Co from Ethiopian ginger is very small and well below the allowable daily intake. Daily intake of toxic metal Cd from Ethiopian ginger is negligible and Pb is none (not present in Ethiopian ginger). Therefore, it can be concluded that Ethiopian ginger is good source of essential metals and free from toxic metal and hence safe for human consumption.

**Table 5:** **Comparison of daily intake of metals from ginger with recommended daily intake and tolerable upper limit of daily intake of metals (NebGuide** 2015 **)**

**Metal**	**Daily intake from ginger (mg/day)**	**Recommended daily intake (mg/day)**	**Allowable upper limit (mg/day)**
**Ca**	2.36	1000	2500
**Mg**	2.00	400	350
**Cu**	0.0195	2	10
**Zn**	0.350	0.150	40
**Mn**	3.71	2	11
**Ni**	0.047	NA	1
**Fe**	34.1	18	45
**Co**	0.0992	NA	NA
**Cr**	0.132	0.120	NE
**Cd**	0.000813	None	0.0714
**Pb**	ND	None	None

NA = data not available. NE = not established. ND = not detected.

### Distribution pattern of metals in soil sample

The soil sample collected from four sampling areas were found to contain detectable metal content of Ca, Mg, Fe, Zn, Cu, Co, Cr, Mn, Ni, and Cd in all the four soil samples and their values are given in Table 4. Among the determined metals, Pb was found to be below the detection limit of the method used in this study. There is significant difference in concentration of different metals within soil sample and appreciable difference in the same metals of different samples. The determined concentration ranges of metals from four soil sampling area are given in Table 4.

As shown in Table 4, the concentration of Fe (21700–46900 mg/kg) in soil exceeds much the concentration of macro-elements, Ca (1770–3580 mg/kg) and Mg (1460–2440 mg/kg), this is due to the presence of excess amount of hematite (Fe_2_O_3_) in the soil.

Concentration of Mn (1760–6470 mg/kg) an essential trace metal in these soils was higher when compared to the micronutrient heavy metals Zn (255–413 mg/kg), Cu (3.76–33.9 mg/kg), Cr (110–163 mg/kg), Co (48.5–159 mg/kg) and Ni (14.1–79.3 mg/kg). On the other hand, level of the toxic heavy metal Cd ranged from 0.24–1.08 mg/kg. The level of Pb, the other tested toxic metal, in soils of all the samples was found to be below the detection limit of the method used in this study. In general, the concentration pattern of metals in soil was decreased as Fe > > Mn > Ca > Mg > Zn > Cr > Co > Ni > Cu > Cd.

### Comparisons of metal levels between ginger and soil sample

Plants absorb whatever is present in the soil medium and therefore the metals are also absorbed and become bio-accumulated in the roots, stems, fruits, grains and leaves of the plant, which may finally be transferred to man in the food chain. The sorption processes of metals by plants is significantly affected by metal level in the soil, soil pH, the presence of competing ligands, the ionic strength of the soil solution and the simultaneous presence of competing metals (Zubillaga et al. 2008).

In this work, comparative study has been established to correlate the metal level of ginger with the soil where it has grown. As it can be seen from Tables 3 and 4, for most elements (Mg, Mn, Zn, Fe (except in Tepi sample), Cu (except in Illubabur sample), Cr, and Cd) the metal levels of ginger was directly proportional to the metal levels of soil where it has grown. Taking the level of Mg as an example in both ginger and soil samples, the Mg level of ginger sample from Illubabur > Tepi > Hadaro > Bombae and the same order is true for Mg level of respective soil sample. This relation partly verifies that the metal content of the plant is a function of the metal level in the soil where it has grown. For the rest three metals (Ca, Ni and Co), the metal levels in some sampling area of ginger were varies non-proportional to levels of metal in the corresponding soil. This non-proportional variation in level of metals in ginger and soil may be resulted from the difference in availability of absorbable form of metals in soil due to difference in soil acidity or the presence of competing ligands. The accumulation factors of the metals from soil to ginger are given in Table 6.

**Table 6:** **Accumulation coefficient of metals from the soil to ginger**

**Metal**	**Sampling sites**			
	**Tepi**	**Bombae**	**Hadaro**	**Illubabur**
**Ca**	0.56	1.23	1.07	1.41
**Mg**	1.23	1.85	1.66	1.68
**Cu**	0.14	0.49	0.37	0.032
**Zn**	0.14	0.12	0.15	0.13
**Mn**	0.060	0.16	0.096	0.086
**Ni**	0.071	0.40	0.32	0.11
**Fe**	0.00094	0.0025	0.0019	0.0019
**Co**	0.048	0.099	0.042	0.017
**Cr**	0.067	0.055	0.080	0.066
**Cd**	0.90	1.58	0.52	0.58

For most metals there was large difference in concentration of the same metals in different ginger sample (for example the concentration of Mg, Mn, Zn, Fe, and Cd of Illubabur and Tepi sample were higher than the other sample sites), this may be attributed to the difference in mineral concentration, the pH and organic matter content of the respective soil. The other probable reason is that the Tepi and Illubabur sample sites are near to the city and may be such differences are observed as a result of higher population and industrial activities in cities and municipalities which lead to higher production of assorted waste than in the rural settlements of Bombae and Hadaro.

### Comparison of levels of metal in ginger of this study with literature values

The comparative study of the metal concentration of ginger determined in this study and reported values of other researchers are presented in Table 7. The Ca content of Ethiopian ginger is a bit higher than that of reported by Devi et al. (2008) and almost comparable to that of reported by Ogunwandea and Olawore (2004). The level of Mg of this study is comparable to those studied in Nigeria (Ogunwandea and Olawore 2004) but much lower than those in India (Devi et al. 2008).

**Table 7:** **Comparison of determined metals concentration (mg/kg, dry mass basis) with reported values**

**Metal**	**Concentration (mg/kg)**	**Country**	**Reference**
**Ca**	1700	India	(Devi et al. 2008)
	2610	Nigeria	(Ogunwandea and Olawore 2004)
	2001-2543	Ethiopia	This study
**Mg**	4210	Nigeria	( Ogunwandea and Olawore 2004)
	9200	India	(Devi et al. 2008)
	2700-4094	Ethiopia	This study
**Mn**	368	Nigeria	( Ogunwandea and Olawore 2004)
	313	India	(Devi et al. 2008)
	184-401	Ethiopia	This study
**Fe**	217	India	(Devi et al. 2008)
	19.4	Saudi Arabia	(Alwakeel 2008)
	41.8-89.0	Ethiopia	This study
**Zn**	72.53	India	(Devi et al. 2008)
	38.5-55.2	Ethiopia	This study
**Ni**	108	Nigeria	(Obiajunwa et al. 2002)
	5.61-8.40	Ethiopia	This study
**Co**	0.32	Saudi Arabia	(Al-Eed et al. 2002)
	2.04-7.58	Ethiopia	This study
**Cu**	4.47	India	(Devi et al. 2008)
	1.10-4.78	Ethiopia	This study
**Cr**	0.47	Pakistan	(Hashmi et al. 2005)
	0.5	Pakistan	(Hashmi et al. 2007)
	6.02 − 10.8	Ethiopia	This study
**Cd**	0.12	Nigeria	(Aiwonegbe and Ikhuoria 2007)
	0.07	Saudi Arabia	(Al-Eed et al. 2002)
	0.38-0.97	Ethiopia	This study
**Pb**	<0.021	Nigeria	(Aiwonegbe and Ikhuoria 2007)
	ND	Ethiopia	This study

ND: Concentration of the tested heavy metal was below the method detection limit.

Mn concentration in Ethiopian ginger is in the range of 184–401 mg/kg and its values reported by Devi et al. (2008) and Obiajunwa et al. (2002) lies in this range. The Fe content reported in Saudi Arabia (Alwakeel 2008) is less than the Fe content in Ethiopian ginger. However, the Fe content reported by Devi et al. (2008) is higher than the result of the present study. The mean concentration of Zn determined in this study is a bit lower than the value determined in India (Devi et al. 2008). The content of Zn in Ethiopian ginger is above the permissible limit set by FAO/WHO in edible plants (27.4 mg/kg). However, according to Bowen and Allaway, the range of Zn in agricultural products should be between 15 to 200 mg/kg (Jabeen et al. 2010).

The Ni concentration in Ethiopian ginger is lower than the Ni content determined in Nigeria (Obiajunwa et al. 2002). The Ni content in Ethiopian ginger is higher than the permissible limit set by FAO/WHO in edible plants (1.63 mg/kg). However, Ni toxicity in human is not a very common occurrence because its absorption by the body is very low (Jabeen et al. 2010).

From Table 7, one can see that the concentration of Cu in Ethiopian ginger is almost the same as the value reported in India (Devi et al. 2008). The Cr content of ginger reported by Hashmi et al. (2005) and Hashmi et al. (2007) are less than the present result. The concentration of Cr in Ethiopian ginger is higher than the permissible limit set by FAO/WHO in edible plants (0.02 mg/kg). Chronic exposure to Cr may result in liver, kidney and lung damage (Jabeen et al. 2010).

The level of Co obtained in Ethiopian ginger is 2.04–7.58 mg/kg, which is higher than the value reported in Saudi Arabia (Al-Eed et al. 2002). The Cd content in Ethiopian ginger is 0.38–0.97 mg/kg, which is higher than the Cd content determined in Nigeria (Aiwonegbe and Ikhuoria 2007) and Saudi Arabia (Al-Eed et al. 2002). The level of Cd in Ethiopian ginger is above the permissible limit set by FAO/WHO in edible plants (0.21 mg/kg). The results of this study reveal that the content of Pb in ginger is below the method detection limit and that assures the low Pb exposure of the farmlands.

The minimal risk levels for hazardous Pb and Cd metals through oral route and its acute effect are 0.0002 mg/kg per day for both metals (Al-Eed et al. 2002). Whereas the human need from ginger is very few grams per day, hence there is no risk from ginger used in the food.

### Analysis of variance

T-tests and analysis of variance (ANOVA) are widely used statistical methods to compare group means. While the independent sample t-test is limited to comparing the means of two groups, the one-way ANOVA can compare the mean of more than two groups of sample. ANOVA uses the F statistic to compare whether the differences between sample means are significant or not (Bereton 2003).

In this study, ginger samples were collected from four different areas and the metal levels of each sample was analysed by FAAS. During the processes of sample preparation and analysis a number of random errors may be introduced in each aliquot and in each replicate measurement. The variation in sample mean of the analyte was tested by using ANOVA, whether the source for variation was from experimental procedure or heterogeneity among the samples (i.e. difference in mineral contents of soil, pH of soil, water, atmosphere; variation in application of agrochemicals like fertilizers, pesticides, herbicides etc. or other variations in cultivation procedures).

The ANOVA results (Table 8) showed that there exist statistically significant differences at 95% confidence level in mean concentrations of all the nine metals except Ni which is expected from variation in experimental procedure. The source for this significant difference between sample means may be the difference in mineral contents of soil or pH of soil which predict the extent of mineral absorption by ginger. For Ni the difference between samples mean is not significant. The variation among results of Ni in four ginger samples should not be attributed to anything more than random error in the analytical procedure.

**Table 8:** **Analysis of variance (ANOVA) between and within ginger samples at 95% confidence level**

**Metal**	**Comparision**	**SD**	**df**	**F**_**calculated**_	**F**_**critical**_	**Remark**
**Ca**	Between samples	255	3	4.96	4.07	Significant difference between sample means
	Within samples	51.4	8			
**Mg**	Between samples	650	3	14.2	4.07	Significant difference between sample means
	Within samples	45.6	8			
**Cu**	Between samples	1.58	3	8.32	4.07	Significant difference between sample means
	Within samples	0.19	8			
**Zn**	Between samples	9.96	3	5.89	4.07	Significant difference between sample means
	Within samples	1.69	8			
**Mn**	Between samples	100	3	13.8	4.07	Significant difference between sample means
	Within samples	7.27	8			
**Ni**	Between samples	1.38	3	3.14	4.07	No significant difference between sample means
	Within samples	0.44	8			
**Fe**	Between samples	21.7	3	5.09	4.07	Significant difference between sample means
	Within samples	4.28	8			
**Co**	Between samples	2.72	3	9.38	4.07	Significant difference between sample means
	Within samples	0.29	8			
**Cr**	Between samples	2.02	3	4.93	4.07	Significant difference between sample means
	Within samples	0.41	8			
**Cd**	Between samples	0.29	3	7.25	4.07	Significant difference between sample means
	Within samples	0.04	8			

where, SD - is standard deviation of between sample and within sample and df - is degree of freedom of between sample and within sample.

### Pearson correlation of metals between ginger and soil samples

In this study, to correlate the effect of one metal concentration on the concentration of the other metal in the same sample and to correlate the effect of the same metal concentration of soil to that of ginger, the Pearson correlation matrices using correlation coefficient (r) for the samples were used. The results are given in Tables 9 and 10. There is high positive correlation for Mg with (Ni, Zn, Mn, Fe and Cr), Cu with (Co and Cd), Zn with (Mn, Cr and Cd), Mn with Cd, Ni with Cr and moderate correlation for Cr with Cd; which may be arise from common anthropogenic or natural sources as well as from similarity in chemical properties. The high negative correlation between Ca and Cu indicate the large absorption of Ca may affect the absorption of Cu in ginger plant. The other metals have weak negative or positive correlation indicating that the presence or absence of one metal affect in lesser extent to the other. In soil samples, there is high positive correlation for all metals except Ca with (all metals except Mn), Co with (all metals except Zn). The low negative or positive correlation of Ca and Co with the other metals in the soil samples may be associated with chemical properties like insoluble carbonates.

**Table 9:** **Correlation matrices for metals in ginger sample (n = 4)**

	**Ca**	**Mg**	**Cu**	**Zn**	**Mn**	**Ni**	**Fe**	**Co**	**Cr**	**Cd**
**Ca**	1									
**Mg**	0.315	1								
**Cu**	−0.907	−0.453	1							
**Zn**	−0.289	0.680	0.310	1						
**Mn**	0.032	0.683	0.120	0.924	1					
**Ni**	0.320	0.866	−0.623	0.311	0.232	1				
**Fe**	0.665	0.916	−0.711	0.443	0.592	0.783	1			
**Co**	−0.376	−0.426	0.731	0.286	0.370	−0.813	−0.438	1		
**Cr**	−0.312	0.751	0.002	0.655	0.399	0.781	0.439	−0.450	1	
**Cd**	−0.554	0.397	0.616	0.941	0.814	0.022	0.115	0.513	0.527	1

**Table 10:** **Correlation matrices for metals in soil sample (n = 4)**

	**Ca**	**Mg**	**Cu**	**Zn**	**Mn**	**Ni**	**Fe**	**Co**	**Cr**	**Cd**
**Ca**	1									
**Mg**	0.428	1								
**Cu**	0.445	0.996	1							
**Zn**	0.252	0.749	0.800	1						
**Mn**	0.706	0.937	0.948	0.737	1					
**Ni**	0.505	0.994	0.997	0.779	0.967	1				
**Fe**	0.461	0.987	0.997	0.839	0.953	0.995	1			
**Co**	0.403	0.313	0.393	0.806	0.477	0.395	0.464	1		
**Cr**	0.052	0.919	0.917	0.789	0.744	0.887	0.908	0.274	1	
**Cd**	0.282	0.942	0.910	0.512	0.808	0.905	0.875	−0.023	0.883	1

As shown in Table 11, one can see that the more levels of metals like Mg, Zn, Mn, Co, Cr and Cd in the soil, the more accumulation of corresponding metals in the ginger. This verifies that the dependence of metal concentration in the plant on metal concentration of respective soil.

**Table 11:** **Pearson correlation coefficient for metals in ginger with soil sample (n = 4)**

**Metal**	**Ca**	**Mg**	**Cu**	**Zn**	**Mn**	**Ni**	**Fe**	**Co**	**Cr**	**Cd**
**r**	−0.812	0.723	0.288	0.857	0.829	0.345	0.459	0.744	0.814	0.766

where, r is the Pearson correlation coefficient between metal level in ginger and metal level in soil.

## Conclusion

An efficient digestion procedure for ginger and soil sample was developed and validated through standard addition (spiking) method and a good percentage recovery was obtained (100 ± 10%) for all minerals identified in ginger.

The levels of metals in ginger determined in this study could be put in the following order Mg (2700–4090 mg/kg) > Ca (2000–2540 mg/kg) > Mn (184–401 mg/kg) > Fe (41.8–89.0 mg/kg) > Zn (38.5–55.2 mg/kg) > Cr (6.02–10.8 mg/kg) > Ni (5.61–8.40 mg/kg) > Co (2.04–7.58 mg/kg) > Cd (0.38–0.97 mg/kg). The non-essential toxic heavy metal, Pb, was found to be below the method detection limit. The results of this work confess that ginger accumulates relatively higher amounts of Mg and Mn among the determined macro- and micronutrients, respectively.

The ANOVA results at 95% confidence level suggest that there were significant difference in the mean concentration of all metals except Ni between the four sampling areas which could be attributed to the difference in mineral contents of soil or pH of soil which predict the extent of mineral absorption by ginger. The soils of the study farmland were found to contain high levels of Fe followed by Mn, Ca, Mg, Zn, Cr, Co, Ni, Cu, Cd. The level of Pb in soils of all sampling sites was found to be below detection limit of the method used in this study.

In general, the levels of most of the metals in the studied soils were found to correlate positively with the levels found in the ginger.
